# Understanding the Physiological Responses of a Tropical Crop (*Capsicum chinense* Jacq.) at High Temperature

**DOI:** 10.1371/journal.pone.0111402

**Published:** 2014-11-03

**Authors:** René Garruña-Hernández, Roger Orellana, Alfonso Larque-Saavedra, Azucena Canto

**Affiliations:** 1 Unidad de Recursos Naturales, Centro de Investigación Científica de Yucatán, Mérida, Yucatán, México; 2 División de Estudios de Posgrado e Investigación, Instituto Tecnológico de Conkal, Conkal, Yucatán, México; Leibniz Institute of Plant Biochemistry, Germany

## Abstract

Temperature is one of the main environmental factors involved in global warming and has been found to have a direct effect on plants. However, few studies have investigated the effect of higher temperature on tropical crops. We therefore performed an experiment with a tropical crop of Habanero pepper (*Capsicum Chinense* Jacq.). Three growth chambers were used, each with 30 Habanero pepper plants. Chambers were maintained at a diurnal maximum air temperature (DMT) of 30 (chamber 1), 35 (chamber 2) and 40°C (chamber 3). Each contained plants from seedling to fruiting stage. Physiological response to variation in DMT was evaluated for each stage over the course of five months. The results showed that both leaf area and dry mass of Habanero pepper plants did not exhibit significant differences in juvenile and flowering phenophases. However, in the fruiting stage, the leaf area and dry mass of plants grown at 40°C DMT were 51 and 58% lower than plants at 30°C DMT respectively. Meanwhile, an increase in diurnal air temperature raised both stomatal conductance and transpiration rate, causing an increase in temperature deficit (air temperature – leaf temperature). Thus, leaf temperature decreased by 5°C, allowing a higher CO_2_ assimilation rate in plants at diurnal maximum air temperature (40°C). However, in CO_2_ measurements when leaf temperature was set at 40°C, physiological parameters decreased due to an increase in stomatal limitation. We conclude that the thermal optimum range in a tropical crop such as Habanero pepper is between 30 and 35°C (leaf temperature, not air temperature). In this range, gas exchange through stomata is probably optimal. Also, the air temperature–leaf temperature relationship helps to explain how temperature keeps the major physiological processes of Habanero pepper healthy under experimental conditions.

## Introduction

Temperature is one of the main conditions that influence plant growth and productivity. According to the Intergovernmental Panel on Climate Change, the global climate is predicted to increase by 2 to 4°C during the next century as a result of increased greenhouse gas concentration in the atmosphere [1]. Global warming may seriously affect plants: e.g., inhibition of photosynthesis [2]–[4] and variation in biomass [5], [6]. Some indicators show that plants could suffer substantial damage as a consequence of high temperature stress. Estimates range up to an average 17% decrease in crop yield for each degree Celsius rise in temperature [7]. However, crop yield is the end result of a chain of physiological events that begins with CO_2_ diffusion into the leaf air space through the stomata, leading to CO_2_ assimilation. This affects the accumulation of biomass and its allocation to different organs, which depends on both the net carbon balance and their programmed growth and expansion [8]. Growing organs are sinks for assimilates which interact with their sources. This interaction is critical to understanding plant responses to environmental factors [9], [10]. Leaf mass decreases at higher temperatures [6]. Nevertheless, the response of boreal and temperate plants is clearly different to that of tropical plants. A given change in temperature has a greater effect on the leaf mass production of tropical species (some changes in the physiological mechanism of plants have been observed) [3], [6]. Thus, growth temperature alters thermal dependence of the photosynthetic rate (temperature acclimation) [3]. In many species, the optimal temperature that maximizes the photosynthetic rate increases with rising growth temperature.

If climate warming increases the frequency of supra-optimal temperatures, it is important to understand the limitations on plant physiology. This is particularly true over the long-term, in order to be able to predict the possible effects of climate change and adapt strategies in agricultural systems and land management to a warmer world. It is imperative to understand how temperature could affect carbon gain [4].

We believe that it is important to investigate the long-term temperature response in a tropical crop. Habanero pepper (*Capsicum chinense* Jacq.) is an excellent study model because it grows to yield a good productivity and quality in wide temperature ranges, and is a profitable crop in the tropical Caribbean and Yucatan Peninsula. It is traded on the international markets, and its fruits and derivatives are used worldwide as condiments, additives, and as the lachrymatory agent in pepper sprays [11]. It is also used as a fungicidal and cytotoxic agent [12]. The aim of this study was therefore to determine the diurnal maximum temperature exposure effects on plant growth analysis and physiological traits in Habanero pepper plants. Three specific questions are addressed here: What are the physiological responses of *C. chinense* to air temperature increase? What is the thermal optimum of this tropical crop under changing environmental conditions? What physiological strategies are involved in achieving physiological plasticity?

## Materials and Methods

### Plant material

Habanero pepper seeds (Seminis) were germinated in 200-well polystyrene trays in a peat moss substrate, in nursery the temperature was ca. 27±3°C and relative humidity (RH) was ca. 80±4%. After 45 days, the seedlings were transplanted to 6 Kg pots containing a soil-peat moss mix (2∶1 v/v) and placed in growth chambers for the experimental phase.

### Growth chambers

Three growth chambers (20 m^3^ each) were placed inside a greenhouse under controlled conditions and were built with transparent glass to allow sunlight to enter. The temperature was controlled with a 5,000 BTU mini-split air conditioner (LG). One diurnal maximum temperature (DMT  =  diurnal air temperature) was calibrated and set per chamber (30, 35, and 40°C) from 8:00 to 18:00 h during the entire experiment. We used these temperatures because 30°C is close to the annual average (27°C) in Yucatan and 35°C is similar to the average maximum temperature in summer. A temperature of 40°C was selected because temperatures above 38°C decrease the physiological mechanism of plants [2]–[6]. In all chambers, the CO_2_ concentration level was measured by sensors (GE Telaire Ventostat 8002), and the average was ca. 380±10 µmol mol^−1^ CO_2_. Plants were naturally illuminated during 11 h day^−1^. The maximum photosynthetic photon flux density (PPFD) at noon was 700 µmol m^−2^ S^−1^. The relative humidity (RH) was ca. 80±4%. The average night temperature was 24±1°C. The latter were measured using data loggers (HOBO H08–004–02; Onset Computer Corp., Bourne, MA, USA) and Quantum sensors (LI–190SB; LI–COR, Lincoln, NE, USA) placed on top and inside of each chamber, as described previously [13].

### Plant growth

Habanero pepper is a short-cycle crop. Thus, the nursery phase was from seed sowing to 45 days after sowing. The flowering phenophase began at 85 days, meaning that the juvenile phenophase was between 46 and 84 days after sowing (before the reproductive phase), and the fruiting phenophase was considered to be when all plants had ripe fruits. As a result, thirty healthy, seedlings were randomly selected 45 days after seed sowing (DASS) and placed in each of the growth chambers. The pots were rotated weekly within the corresponding chamber to avoid edge effects. Plant traits such as leaf area, dry mass (DM), dry mass proportion (DM%), relative growth rate (RGR), specific leaf area (SLA), net assimilation rate (NAR), leaf area ratio (LAR), leaf weight ratio (LWR) and stomata number (abaxial and adaxial) were determined by a series of four destructive harvests. Harvest 1 (T0) was performed at the onset of treatments (45 DASS); harvest 2 (T1) at juvenile plant stage (65 DASS); harvest 3 (T2) at flowering phenophase (110 DASS), and harvest 4 (T3) at fruiting phenophase (130 DASS). In each harvest, 5 plants per treatment were randomly selected. RGR was determined for the period including the first harvest (T0) up to the next harvest (T1, T2 and T3 respectively). Total leaf area of each plant was taken with a surface area meter (LICOR, LI-3100, Nebraska, USA). All samples were dried in an oven at 65°C until constant weight was reached and then dry mass (DM) was determined. Relative growth rate (RGR), leaf area ratio (LAR), specific leaf area (SLA), net assimilation rate (NAR) and leaf weight ratio (LWR) were calculated as described by Hunt et al. [14]. These five parameters are defined and related in the following way:
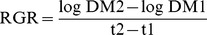


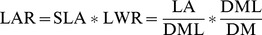


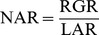



Where DM is total dry mass per plant, t is time, LA is total leaf area per plant and DML is total leaf dry mass per plant.

### Stomatal counts

In each phenophase (juvenile, flowering, and fruiting), 5 plants were selected and stomata number was counted per plant at three different levels (top, middle, and bottom), taking counts for both abaxial and adaxial layers. Three samples were measured per level. The stomata were not counted on the leaves themselves because they are not directly visible under an optical microscope. Therefore, for sampling, a glue drop of ethyl cyanoacrylate (Kola Loka) was applied on a slide. The leaf was placed over it (between the central leaf vein and leaf border) and pressed for about 30 seconds, thereby obtaining an impression on the slide. 15 optical fields per impression were photographed under a microscope at 40X (Leica BME H22), and stomata number was calculated per mm^2^.

### Gas exchange analyses

Gas exchange analyses were carried out inside each growth chamber at relevant growth conditions with a non-invasive method, using a portable infrared gas analyzer system (IRGA; LICOR, LI–6400, Nebraska, USA). At 07:00, 09:30, 12:00, 14:30 and 17:00 h, fifteen fully expanded young leaves from each treatment were placed in the gas-exchange leaf chamber of the IRGA LI–6400 (Five plants per treatment were exclusively for gas exchange analyses). CO_2_ assimilation rate (*A_N_*), stomatal conductance (*g*_s_), intercellular CO_2_ concentration (*C_i_*) and transpiration (*E*) were estimated under the growth chamber conditions described above. Intercellular CO_2_/atmospheric CO_2_ (*C_i_*_/_ *C_a_*) ratio and temperature deficit (air temperature – leaf temperature; *T_air_* – *T_leaf_*) were subsequently calculated.

*A*–*C*_i_ curve analyses were carried out inside each growth chamber. At 7:00 h, fully expanded apical leaves were placed in the LI-6400 leaf chamber. In this case, leaf temperature (not DMT) was used for estimations. The analyses were repeated on five different leaves for each treatment. The gas-exchange response to CO_2_ was measured from 0 to 2000 µmol mol^−1^ CO_2_. The maximum photosynthetic rate (A_max_) was estimated at a PPFD of ca. 1200 µmol m^−2^ s^−1^ using equations developed by von Caemmerer and Farquhar [15]. Stomatal limitation (*l*), which is the proportionate decrease in light-saturated net CO_2_ assimilation attributable to stomata, was calculated according to Farquhar and Sharkey [16] as *l* = (*A*0–*A*1)/*A*0, where *A*0 is the *A* at *C*_i_ of 360 µmol mol^−1^ and *A*1 is *A* at ca of 360 µmol mol^−1^.

### Experimental design

A completely random design was used with 30 plants per growth chamber. Data were examined by ANOVA and treatment means were compared using Tukey's HSD test at P<0.05 (Statistic Six Sigma, Release 7, StatSoft). An arcsine square root transformation was used to adjust the normality of the data percentages (in dry mass proportion).

## Results

**Figure 1:**
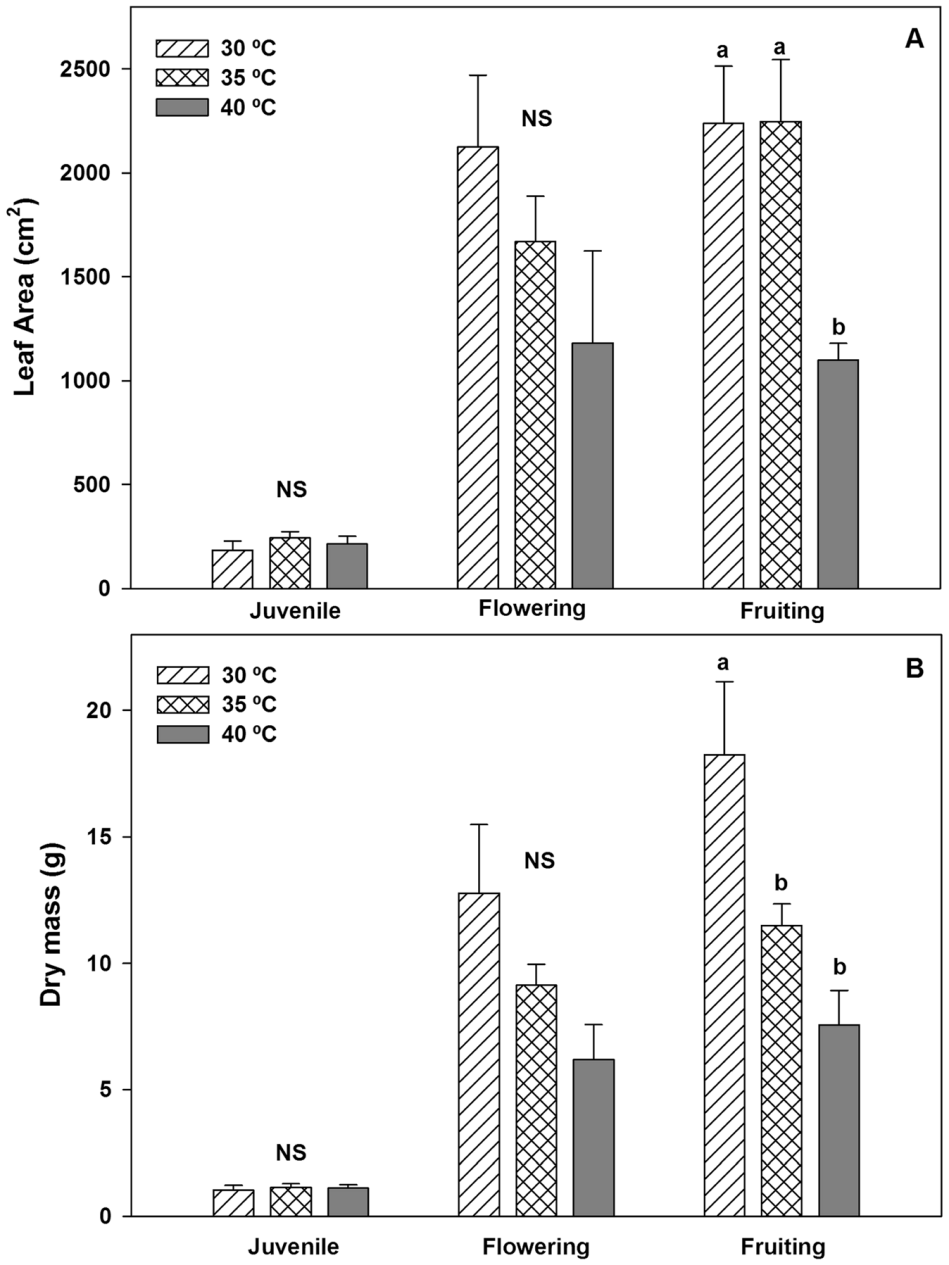
Leaf area and dry mass of Habanero pepper plants. Figure 1. A) Leaf area and B) dry mass of Habanero pepper plants in different phenological stages at three maximum diurnal temperatures (T_air_ = 30, 35 and 40°C). Data are means ± SE. Different letters in the same phenology stage represent statistically significant differences (Tukey, α = 0.05). *n* = 5.

High temperature affects plant growth. In this study, both leaf area and dry mass of Habanero pepper plants did not exhibit significant differences in juvenile and flowering phenophases. However, in the fruiting phenophase, the leaf area and dry mass of plants grown at 40°C DMT were 51 and 58% lower than plants at 30°C DMT respectively (Figures 1A and 1B).

**Figure 2:**
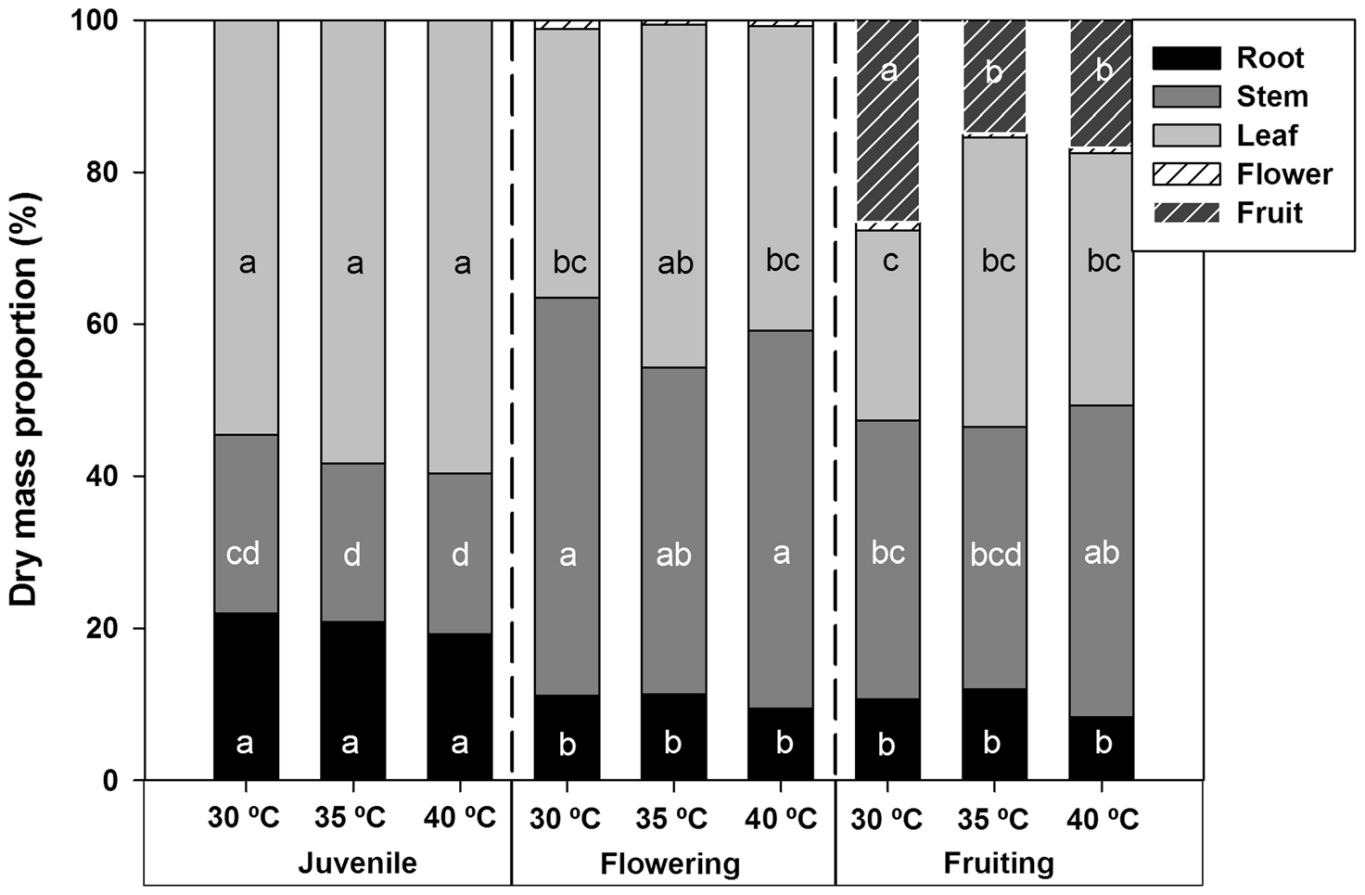
Dry mass proportion in different phenophases. Figure 2. Average dry mass proportion of Habanero pepper plants in different phenological stages at three maximum diurnal temperatures (T_air_ = , 35 and 40°C). Different letters in the same organ represent statistically significant differences (Tukey, α = 0.05). *n* = 5.

The dry mass proportion per organ (DM%) is an indicator of how temperature affects each plant organ. In this case, dry mass proportion of Habanero pepper plants changed in each phenology stage. However, DM% of roots, stems, leaves and flowers did not exhibit significant differences between treatments. But, dry mass proportion in fruiting at 30°C was at least 37% higher than others treatments (Figure 2).

**Figure 3:**
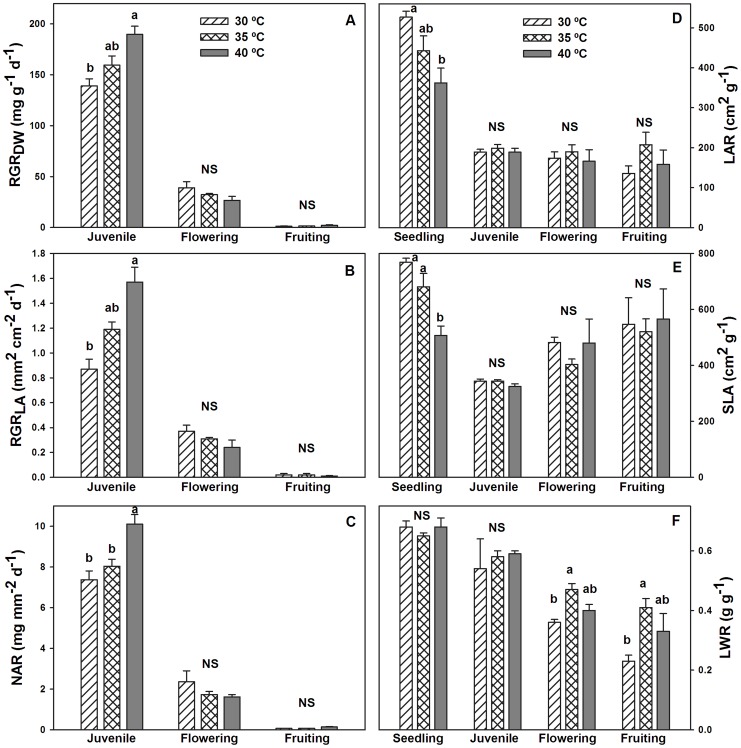
Growth parameters of Habanero pepper plants. Figure 3. A) Relative growth rate (dry mass); B) Relative growth rate (leaf area); C) Net assimilation rate; D) Leaf area ratio; E) Specific leaf area; and F) Leaf weight ratio of Habanero pepper plants at three maximum diurnal temperatures (T_air_ = 30°C, 35°C and 40°C) by phenological stage. Data are means ± SE. Different letters in the same phenology stage represent statistically significant differences (Tukey, α = 0.05). *n* = 5.

Growth parameters show the use of resources by the plant. In all treatments, relative growth rate based on dry mass (RGR_DW_), relative growth rate based on leaf area (RGR_LA_) and net assimilation rate (NAR) did not differ during flowering and fruiting stages. However, in juvenile plants at 40°C DMT, these three parameters were 37, 44 and 37% higher than plants at 30°C DMT respectively (Figures 3A, 3B and 3C). Leaf area ratio (LAR) and the specific leaf area (SLA) did not exhibit significant differences in juveniles, or in flowering and fruiting stages. Both were significantly reduced by 32 and 34% respectively when temperature was increased from 30 to 40°C during seedling stages (Figures 3D and 3E). In contrast, leaf weight ratio (LWR) was not significant in either seedling or juvenile stages, but increased by 23 and 44%, respectively in flowering and fruiting stages when temperature was increased from 30 to 35°C (Figure 3F). Probably the results of juvenile plants were a consequence of few days within the chamber.

**Figure 4:**
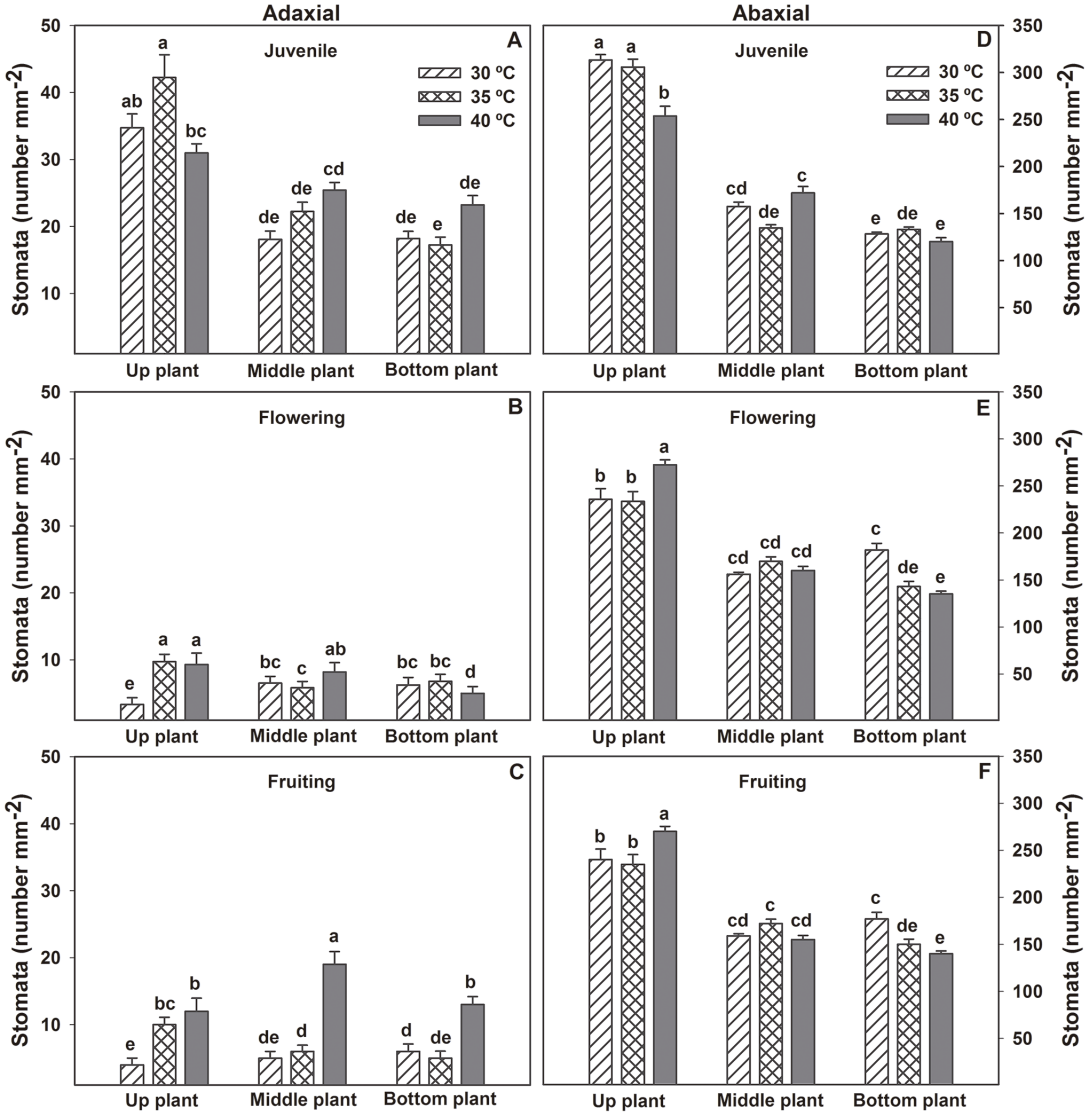
Stomata number of Habanero pepper plants. Figure 4. Stomata number (adaxial and abaxial) at three height levels of plants (top, middle, and bottom) and different phenological stages, A) Adaxial juvenile; B) Adaxial flowering; C) Adaxial fruiting; D) Abaxial juvenile; E) Abaxial flowering and F) Abaxial fruiting) of Habanero pepper plants at three maximum diurnal temperatures (T_air_ = 30, 35 and 40°C). Data are means ± SE. Different letters represent statistically significant differences (Tukey, α = 0.05). *n* = 5. The scale of graphs on the left is different to the graphs on the right.

Stomata permit gas exchange between the inside and outside of plants. An increase in stomata number could change the gas-exchange rate. The occurrence of amphistomatous leaves is not unusual for pepper plants, although it has not been reported for this species. Therefore, amphistomatous leaves are reported in this study on Habanero pepper plants. However, in juvenile, flowering and fruiting stages the stomatal number in the adaxial side was at least 81, 95 and 87% lower than on the abaxial side respectively. The stomatal density on abaxial sides decreased from juvenile to flowering stage. Additionally, stomatal number decreased from the upper leaf section to the lower leaf section (Figure 4).

**Figure 5:**
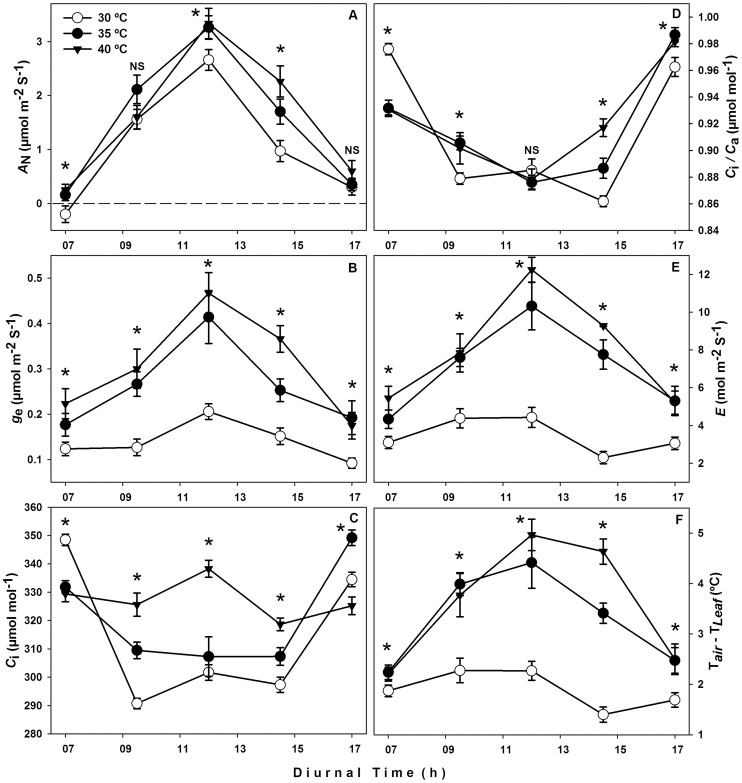
Physiological responses to increased air temperature. Figure 5. A) CO_2_ Assimilation rate (*A*_N_); B) Stomatal conductance (*g*_e_); C) Intercellular CO_2_ concentration (*C_i_*); D) Ratio of intercellular CO_2_/atmospheric CO_2_ (*C_I/_C_a_*); E) Transpiration (*E*); and F) Temperature deficit (*T_air_* – *T_leaf_*) of Habanero pepper plants at three maximum diurnal temperatures (T_air_ = 30°C, 35°C and 40°C). Data are means ± SE. *n* = 15. NS: no significance, *: significant (ANOVA, P<0.05).

Physiological parameters are fundamental for understanding plants' responses to the environment. In this study, plants at 40°C DMT had a net CO_2_ assimilation rate that was 20 and 57% higher than those at 30°C DMT at 12:00 and 14:30 h respectively (Figure 5A), when photosynthetic photon flux density was at its maximum (PPFD). In addition, stomatal conductance at 40°C DMT was 57% higher than in plants at 30°C DMT at 12:00 h (Figure 5B). Intercellular CO_2_ (*C_i_*) in plants at 40°C DMT was higher than in plants at 30 and 35°C DMT between 9:00 and 14:30 h (Figure 5C). At noon, there was no difference in the intercellular CO_2_/atmospheric CO_2_ ratio between treatments, but at 7:00 and 17:00 h the plants measured in all treatments were close to 1 (Figure 5D). Transpiration rate increased in higher temperature treatments (35 and 40°C DMT) during the course of the day (Figure 5E) and was very similar to stomatal conductance. Temperature deficit (*T_air_* – *T_leaf_*) in plants at 40°C reached 5°C at 12:00 h and 4.6°C at 14:30 h; ca. 2.7 and 3.3°C higher than plants at 30°C. Although air temperature in each chamber was 30, 35 and 40°C DMT, the leaf temperature was ca. 27.8, 30.5 and 35°C, respectively. This result was significant in all treatments (Figure 5F).

**Figure 6:**
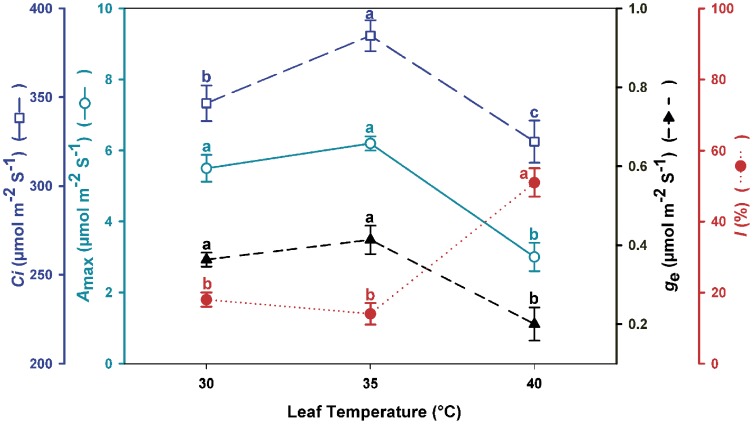
Physiological responses to increased leaf temperature. Figure 6. Intercellular CO_2_ concentration (*C_i_*), maximum photosynthetic rate (*A*_max_), stomatal conductance (*g*_e_) and stomatal limitation (*l*) of Habanero pepper plants at three leaf temperatures (T_leaf_ = 30°C, 35°C and 40°C). Data are means ± SE. Different letters in the same parameter represent statistically significant differences (Tukey, α = 0.05). *n* = 5.

In the *A*–*C*_i_ curves, the photosynthetic system of leaves is gradually saturated with CO_2_, providing a measure of physiological plasticity of the plant in different environments. The *A*–*C*_i_ curve analyses conducted on Habanero pepper plant leaves showed that at a leaf temperature of 40°C (not DMT), there was a decrease in the maximum photosynthetic activity (52%), intercellular CO_2_ (15%) and stomatal conductance (50%) as a result of a 33% increase of in stomatal limitation (Figure 6).

## Discussion

An increase in maximum temperature above the thermal optimum should have a negative effect on physiological performance. For example, photosynthesis [17], [18], respiration [19], plant growth [14] and reproductive processes such as flowering and fruit set may be especially sensitive to high temperatures [20], [21]. However, the thermal optimum is higher in a tropical plant than in a temperate plant, because tropical plants are high temperature adapted species [22].

According to our results, the thermal optimum for leaf temperature in Habanero pepper should be below 35°C (Figure 1–6). Although the decrease in leaf temperature should be between 1 or 2°C with respect to air temperature [23], according to our results, a rise in diurnal maximum temperature increased the transpiration rate (Figure 5E) with the effect that this kept leaf temperatures close to the thermal optimum range for Habanero pepper (between 30–35°C). This caused the temperature to decrease to 5°C below the air temperature (Figure 5F). Thus, we consider that the *T_air_* – *T_leaf_* relationship describes the maintenance of healthy major physiological processes (i.e. photosynthesis), causing a rise in both stomatal conductance (Figure 5B) and intercellular CO_2_ concentration (Figure 5C), as well as a higher CO_2_ assimilation rate than observed at low temperatures (Figure 5A).

Inhibition of photosynthesis by heat stress commonly occurs in tropical and subtropical plants [22]. However, the optimal temperature for photosynthesis varies to a limited extent depending upon the conditions for growth [24]. Furthermore, some growth parameters such as leaf area (Figure 1A) and dry mass (Figure 1B) exhibited lower values at higher diurnal temperature in reproductive processes (flowering and fruiting). The latter was attributed to an increase in abortion of both flowers and fruits (Figure 2). We observed that DMT Habanero pepper plants at 40°C decreased their fruit set three-fold with respect to values obtained for plants at 30°C DMT [25]. Moreover, flower abortion of plants at 40°C DMT increased two-fold when compared to plants at 30°C DMT, probably due to hormonal imbalance as a consequence of elevated temperature [25]. Hormonal activity has an effect on abortion of flowers and fruits in reproductive phenophases [26]. This suggests that an elevated diurnal maximum air temperature does not have a negative effect on the photosynthetic apparatus in a tropical plant like Habanero pepper (Figure 5A), but could have a negative effect on hormonal activity and diminish some major economic traits in tropical crops. However, if air temperature is very high (above 40°C) and the plant's regulatory mechanism could not maintain the leaf temperature at its thermal optimum, then a decrease in intercellular CO_2_ concentration, maximum photosynthetic rate and stomatal conductance (*g*_e_) would be caused by an increase in stomatal limitation, as was observed in the results (Figure 6).

The purpose of taking data measurements over the whole day was to identify at what point the CO_2_ assimilation rate decreases as the air temperature rises. About 30% of the carbohydrate formed in C_3_ photosynthesis is lost through photorespiration [27] and the loss increases with temperature. However, in Habanero pepper under the established temperature intervals, CO_2_ assimilation rate was always observed during diurnal measurements (Figure 5A).

On the other hand, elevated temperature had no significant influence on stomata number. However, both leaf position on the plant (bottom, middle, and top) as well as leaf side (abaxial and adaxial) showed significant differences in stomata number (Figure 4). This suggests the influence of other factors, such as light captured by leaves at the three different levels (top, middle, and bottom) [28], could have had a major environmental effect.

## Conclusions

A change in maximum diurnal temperature tends to modify a chain of physiological events. In this study we showed that an increase in diurnal air temperature raised both stomatal conductance and transpiration rate in Habanero peppers, causing an increase in temperature deficit (air temperature – leaf temperature). Leaf temperature decreased by 5°C, allowing a higher CO_2_ assimilation rate in plants at diurnal maximum air temperature (40°C). However, in *A*–*C*_i_ curves when leaf temperature was set at 40°C, physiological parameters decreased due to an increase in stomatal limitation. We showed that the thermal optimum range in a tropical crop such as Habanero pepper is between 30 and 35°C (leaf temperature, not air temperature). In this range, gas exchange through stomata is probably optimal in tropical species for good performance. Unexpectedly, CO_2_ assimilation rate was always present in our experiments. Although an increase in air temperature enhanced photosynthesis values at diurnal measurements, this was not reflected in plant production (leaf area, dry mass and relative growth rates). Thus, photosynthate surplus is probably channelled and used in other metabolic pathways instead of producing biomass. In consequence, the physiological process in plants is maintained, avoiding damage due to high temperatures.
